# The role of extracellular matrix in tumour angiogenesis: the throne has NOx servants

**DOI:** 10.1042/BST20200208

**Published:** 2020-11-05

**Authors:** Amir M. Alsharabasy, Sharon A. Glynn, Abhay Pandit

**Affiliations:** 1 CÚRAM, SFI Research Centre for Medical Devices, National University of Ireland, Galway, Ireland; 2 Discipline of Pathology, Lambe Institute for Translational Medicine, School of Medicine, National University of Ireland, Costello Road, Galway, Ireland

**Keywords:** extracellular matrix, metastasis, nitric oxide, nitric oxide Extr tumour angiogenesis

## Abstract

The extracellular matrix (ECM) dynamics in tumour tissue are deregulated compared to the ECM in healthy tissue along with disorganized architecture and irregular behaviour of the residing cells. Nitric oxide (NO) as a pleiotropic molecule exerts different effects on the components of the ECM driving or inhibiting augmented angiogenesis and tumour progression and tumour cell proliferation and metastasis. These effects rely on the concentration of NO within the tumour tissue, the nature of the surrounding microenvironment and the sensitivity of resident cells to NO. In this review article, we summarize the recent findings on the correlation between the levels of NO and the ECM components towards the modulation of tumour angiogenesis in different types of cancers. These are discussed principally in the context of how NO modulates the expression of ECM proteins resulting in either the promotion or inhibition of tumour growth via tumour angiogenesis. Furthermore, the regulatory effects of individual ECM components on the expression of the NO synthase enzymes and NO production were reviewed. These findings support the current efforts for developing effective therapeutics for cancers.

## Introduction

**Figure 1.**
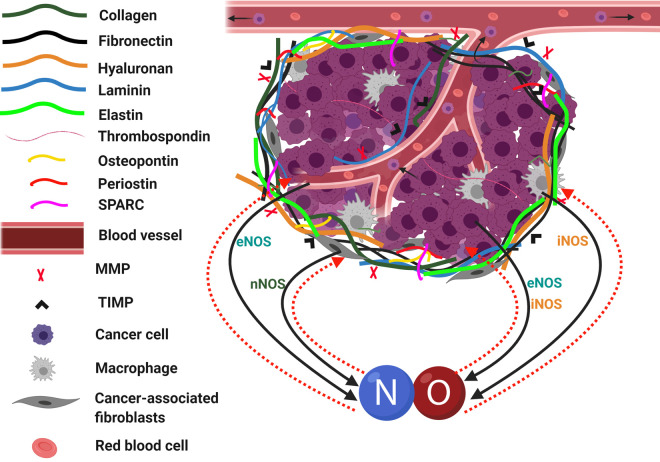
Composition of tumour tissue and how NO is generated and interacts with its constituents. The tissue consists mainly of cancer cells, tumour-associated macrophages, cancer-associated fibroblasts, and endothelial cells lining the blood vessels through which these cells get their nutrients, and the tumour cells can metastasize. Nitric oxide (NO) is produced from these cells via different isoforms of NO synthase and works as a signalling molecule for activating various transduction pathways. The extracellular matrix (ECM) of tumour tissue is produced by the resident cells and generally consists of three main categories of proteins: (1) structural proteins: such as collagen, fibronectin, hyaluronan, laminin and elastin; (2) matricellular proteins: such as thrombospondin, osteopontin, periostin and SPARC proteins; (3) MMPs and tissue inhibitors of metalloproteinases (TIMPs). There are mutual interactions between NO, the ECM components and cells for modulating the tumour growth, angiogenesis and metastasis.

Nitric oxide (NO) exerts multiple effects on tumour biology, where it can be both pro- and anti-tumorigenic depending on the NO dose and duration of exposure. These effects are mediated by the different components of the tumour tissue including the different cells residing in the tumour tissue, which produce and are sensed by NO and the multiple constituents of the extracellular matrix (ECM). In human, NO is mainly synthesized in the tumour tissue via three nitric oxide synthase (NOS) enzymes: endothelial NOS (eNOS), inducible NOS (iNOS) and neuronal NOS (nNOS), all of which can play a role in tumorigenesis including tumour proliferation, angiogenesis and tumour metastasis (Figure 1). Tumours, including breast cancer [1], hepatocellular [2,3], colorectal cancer [4] overexpressing iNOS have been found to display increased angiogenesis. Similarly, eNOS has been shown to modulate angiogenesis in several tumour types, including hepatocellular cancer [5], colorectal cancer [6], and glioblastoma [7]. Herein, the influence of NO can be either: (1) on individual cascades within the cancer cells through either the activation or repression of expression of specific cytokines, or (2) via its influence on specific structural and matricellular proteins on ECM-degrading enzymes. NO regulation of angiogenesis is a key factor in facilitating tumour growth and metastasis. While the focus of this review is on the role of NO in the regulation of the ECM and angiogenesis, in addition NO influences tumour biology in several ways. NO is a key DNA mutagen and contributes to the accumulation of DNA mutations. NO is associated with accumulation of mutations in p53 in colorectal cancer [8] and K-Ras in hepatocellular carcinoma [9]. NO also contributes to tumorigenesis by induction of cell migration and invasion via regulation of epithelial to mesenchymal transition (EMT) [10,11]. and activation of matrix metalloproteases that facilitate tumour invasion and extravasation [12]. Additionally, NO regulates response to chemotherapy and thus influences patient outcomes [13,14].

Cancer tissues are composed of (i) cellular components including tumour cells, stroma which includes endothelial, smooth muscle, immune cells, epithelial fibroblasts and myofibroblasts along with different types of mesenchymal cells and (ii) non-cellular part comprising of the ECM proteins [15,16]. The interactions between non-tumour and tumour cells, in addition to the aberrant activation of specific ECM proteins and the exposure to the secreted molecules within the tumour microenvironment, leads to the acquisition of cancer-associated phenotypes [16,17]. For instance, in healthy tissues, the ECM dynamics, including their production, degradation and further remodelling, are tightly regulated via specific mechanisms controlled by the cells [18]. However, in the case of cancer, deregulation of these signals and mechanisms takes place leading to a change in the behaviour of the residing cells with corresponding abnormal ECM dynamics and disorganization of the ECM architecture [19]. The constituents of the ECM vary according to the type and locations of the tumour tissue. However, in general, these include: (i) structural proteins: collagens (mainly type I, II, IV, VIII, XV, XVIII, and XIX), elastin, laminins (111, 332, and 511), mucin, hyaluronic acid (HA) with different average molecular weights, fibronectin, fibrillin, fibulin, syndecan, glypican, vitronectin, aggrecan, perlecan, versican, decorin, lumican, testican, nidogen and biglycan [20–22] (Figure 1). (ii) matricellular proteins: thrombospondin, small integrin-binding ligand N-glycosylated (SIBLINGs) mainly osteopontin, periostin, secreted protein acidic and rich in cysteine (SPARC), CCN proteins, and Tenascin-C [23–25]. (iii) ECM-remodelling enzymes and their inhibitors: a wide variety of enzymes including different matrix metalloproteinases (MMPs), lysyl oxidase, HA synthases and hyaluronidase [26–28]. The ECM enzymes are the primary regulators for the ECM remodelling, and the changes in these enzymes take place at the transcriptional, translational, and post-translational levels [18,29].

In this review, we examine the role of NO in the modulation of ECM and its implications for tumour angiogenesis. Furthermore, the recent studies on the effects of the ECM components on NOS expression and NO production in tumours are comprehensively analysed with highlighting the required research for the complete understanding of these interactions. This will help get a more in-depth idea on how the tumour tissues develop and facilitate the screening and development of more effective treatments for cancers.

## ECM remodelling enzymes and nitric oxide

For the metastasis of cancer cells, degradation of the stromal ECM and basement membrane is mediated by MMPs to facilitates tumour access to the vascular and lymphatic systems [30,31]. MMPs are considered the main class of proteolytic enzymes involved in cancer progression, and their roles have been discussed previously [32–34]. These enzymes are secreted by various cell types, with specific activities in certain tissues, where a number of ECM components regulate the expression of the MMP genes and proteins as well as their enzymatic activation [28,35,36]. NO is one of these regulating factors, which, based on its concentration and the duration of exposure, can regulate different MMPs at both the transcriptional and translational levels. The recent findings on NO regulation of MMPs are not solely limited to tumour tissues but are also seen in healthy tissues. For example, the expression of MMP-9, known to correlate with lung injury and epithelial cell death was found to be induced by NO in a soluble guanylyl cyclase (sGC) dependent manner and to be suppressed by Wilms Tumour 1 transcription factor [37,38]. Moreover, a positive correlation between the levels of NO and MMP-9 was found in the serum of breast cancer patients and is believed to be associated with the development of metastasis [39]. The recent findings on NO regulation of MMPs are summarized in Table 1.

**Table 1:** Effects of NO on the MMPs involved in different types of cancers

Enzyme	Expressing cells/tissue	NO source	Effects of NO	Cancer type	Ref
MMP-1	The Melanoma cell line (C32TG)	S-nitrosoN-acetylpenicillamine (SNAP) (62.5–1000 µM)	Enhanced MMP-1 mRNA and protein expression enzyme with up-regulation of its promoter activity via the mitogen-activated protein kinase (MAPK) (extracellular signal-regulated kinase (ERK) and p38) pathways	Melanoma	[40]
		Induction of iNOS expression			
	The Melanoma cell line (Mewo)	SNAP (62.5–1000 µM)	Enhanced MMP-1 mRNA and protein expression		
	Human uterine cervical fibroblasts	Sodium nitroprusside (SNP) (0.1–100 µM) & diethylenetriamine NONOate (DETA-NONOate) (1–100 µM)	Enhanced pro-MMP-1 mRNA expression and protein synthesis	Uterine cervical cancer	[41]
		Induced iNOS expression	Enhanced MMP-1 protein production, which is inhibited in the presence of the NOS-inhibitor N(gamma)-nitro-l-arginine methyl ester (l-NAME).		
	The glioma cell lines (U-87MG and T98G)	SNP (100 µM)	Enhanced MMP-1 mRNA and protein expression with increased glioma cell movement	Glioma	[42]
MMP-2	The fibrosarcoma cell line (HT1080)	SNP (1–200 µM)	No effects on the pro-MMP-2 levels and tumour invasion	Fibrosarcoma	[43]
	The ovarian cancer cell lines (SK-OV-3 and OVCAR-3)	Spermine-NO complex and DETA-NONOate (1000 µM)	-Decreased MMP-2 release with higher efficiency in the case of DETA-NONOate via the Guanosine 3′,5′-cyclic monophosphate (cGMP) pathway, but with no effects on the levels of proMMP-2. These results accompanied decreased cell penetration through Matrigel. -Only NO from DETA-NONOate caused decreased MMP mRNA expression in SK-OV-3 cells, but both caused the decreased activity of the released MMP. -Neither of the NO donors affected mRNA expression and activity of the released MMP in OVCAR-3 cells. -These results were correlated with decreased cell penetration through Matrigel.	Ovarian cancer	[44]
	The colon cancer cell line (WiDr)	SNP (15.6 µM)	A 12-fold increase in MMP-2 mRNA expression following the treatment for 8 h, then declined, with decreased expression of TIMP-1/2, and promoted MMP activity. This improved activity accompanied by cell migration was regulated via cGMP- protein kinase G (PKG)-ERK-1/2 dependent mechanism.	Colon cancer	[45]
	The lung cancer cell line (A549)	Gaseous NO (1–10 µM)	-Enhanced MMP-2 mRNA and protein expression owing to the promoted iNOS expression along with the decreased expression of TIMP-2, with improved cell migration. This is mediated by the nuclear translocation of NF-κB and c-Jun. -Similar results were obtained following the injection of the NO-induced cells in mice with enhanced metastasis of the lung cells and MMP activity.	Lung cancer	[46]
	The carcinoma cell lines from primary head (H) and neck (N) (HN18 & HN30) and metastatic sites (HN18 & HN30)	Diethylamine NONOate (DEA-NONOate) (6500 and 8500 µM)	-Significant increase of MMP-2 activity in HN18 and HN30, decrease in HN31, but with no changes in HN17, with a corresponding decrease in cell viability. These results were not correlated with the inhibition of cell invasion through Matrigel.	Head and neck squamous cell carcinoma (HNSCC)	[47]
MMP-3	C32TG	SNAP (500 µM)	Enhanced MMP-3 mRNA expression.	Melanoma	[40]
	WiDr	SNP (15.6 µM)	Enhanced MMP-3 mRNA expression following the treatment for 8 h with increased cell proliferation.	Colon cancer	[45]
MMP-9	HT1080	SNP (1–200 µM)	No effects on the pro-MMP-9 levels and tumour invasion.	Fibrosarcoma	[43]
	SK-OV-3 and OVCAR-3	Spermine-NO complex and DETA-NONOate (10–1000 µM)	-Inhibited MMP-9 activity in the case of SK-OV-3 cells only. -Despite the decreased release of MMP from OVCAR-3, there were no effects of the NO donors on its activity. -These results were associated with decreased cell penetration through the Matrigel.	Ovarian cancer	[44]
	WiDr cells	SNP (15.6 µM)	Eightfold enhanced MMP-9 mRNA expression following the treatment for 8 hours, which then declined, accompanied by decreased expression of TIMP-1/2 and promoted MMP activity. This improved activity accompanied with enhanced cell migration were regulated via cGMP-PKG-ERK-1/2 dependent mechanism.	Colon cancer	[45]
	A549 cells	Gaseous NO (1–10 µM)	No changes in the MMP-9 expression following iNOS induction, but the activity was improved in mice after injection of the NO-induced cells with enhanced metastasis.	Lung cancer	[46]
	Human umbilical vein endothelial cells (HUVECs) and NCI-H157 squamous carcinoma cells co-culture and HUVECs and NCI-H522 adenocarcinoma cells co-culture.	Spermine-NONOate (500 and 1000 µM) and 3-morpholino-sydnonimine (20, 200 and 2000 µM)	-Reduced MMP-9 activation in the ECs once NO levels are elevated at the cultured cell interface with distinct locations with Cav-1 within the cells. -The MMP activity is restored following treatment with the aminoguanidine as an iNOS inhibitor with colocalization of MMP-9 and Cav-1 and formation of new sprouting structures. -Both the NO donors inhibited the proenzyme and activated form of MMP after the direct incubation of NO-donor with purified MMP-9 standards	Lung cancer	[48]
	HN18/HN17, HN30/HN31	DEA-NONOate (6500 and 8500 µM)	No effects on the MMP-9 activity.	HNSCC	[47]
MMP-10	C32TG	SNAP (500 µM)	It enhanced MMP-10 mRNA expression.	Melanoma	[40]
	Mewo cells				
	WiDr cells	SNP (15.6 µM)	Enhanced MMP-10 mRNA expression following the treatment for 8 hours with increased cell proliferation.	Colon cancer	[45]
MMP-13	C32TG	SNAP (500 µM)	It enhanced MMP-13 mRNA expression.	Melanoma	[40]

The regulation of MMPs by NO also takes place at the level of the ECs and modulation of angiogenesis. For example, Membrane Type 1-MMP (MT1-MMP) degradation of type I collagen at the cell membrane of HUVECs was activated by DETA-NONOate and accompanied with enhanced cell migration and tube formation *in vitro* [49]. Similar findings were seen with human microvascular endothelial cell line [50]. Additionally, intussusceptive angiogenesis responsible for the expansion of microvasculature in inflammatory bowel disease was regulated by MT1-MMP and NO, where MT1-MMP cleaved thrombospondin-1 (TSP-1), and the C-terminal fragment of TSP-1 activated eNOS expression and NO production via binding to CD47/α_V_β_3_ integrin [51]. Besides, the treatment of bovine aortic endothelial cells (BAECs) with NO donor DEA-NONOate caused enhanced mRNA expression via the cGMP pathway and proteolytic cleavage of pro-MMP-13, further implicating a role for NO in angiogenesis [52]. These observations refer to the roles played by NO in the regulation of expression of the different MMPs involved in tumour angiogenesis as well as modulating their activities. These functions are dependent on the concentration of NO or the NO releasing compound, duration of exposure and tumour type, where, in certain tumours, some MMPs are activated, while others are not affected by NO.

## Influence of the ECM and NO on tumour angiogenesis

**Figure 2.**
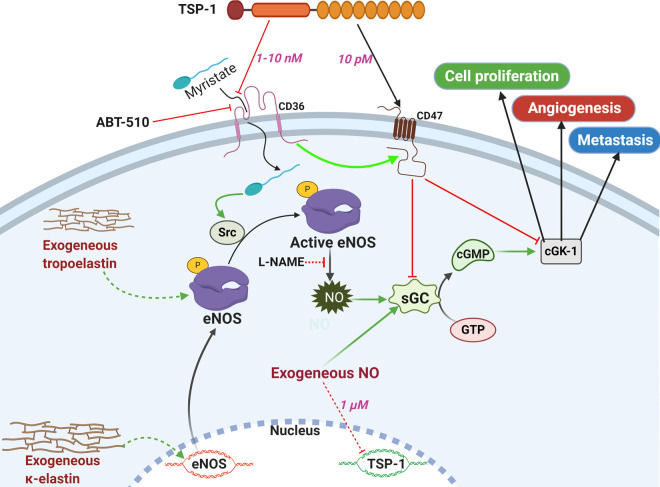
The synthetic pathway of NO in ECs, inducers, inhibitors, the roles played and the effects of different components of the ECM. The exogenous κ-elastin enhances the mRNA expression of eNOS in the ECs [73], and tropoelastin improves the protein expression for NO production, which is inhibited by L-NAME [74]. The TSP-1-mediated inhibition of NO synthesis and downstream effects is through two main mechanisms: (1) The high concentrations of TSP-1 and its peptide mimetics, ABT-510, via interaction with CD-36, inhibit the translocation of myristate to the cytoplasm, thus inhibit the membrane translocation of the Src responsible the activation of eNOS and generation of NO [68,70]. (2) At low concentration, and via its binding to CD47, TSP-1 inhibits the NO-mediated activation of sGC, cGMP generation from Guanosine triphosphate (GTP), and activation of cGMP-dependent protein kinase (cGK-1) responsible for the enhanced cell proliferation, angiogenesis and metastasis [71,72]. The exogenous NO at a concentration of 1 µM only can decrease the protein expression of TSP-1 in the ECs, while activates sGC [67]. The scheme was created in Biorender.com.

For sustained tumour growth, the demands for nutrients and oxygen, as well as for waste exchange, makes it essential for angiogenesis and lymphangiogenesis to be activated, so the tumour can grow, and metastasize to facilitate cancer progression [53,54]. The different components of the ECM play different roles in regulating the sprouting of new blood vessels during different stages of angiogenesis. For instance, different peptide fragments from individual ECM components may have a stimulatory role (e.g. elastin), while others have inhibitory (e.g. thrombospondin) functions during angiogenesis [21,55,56]. Under the effect of several angiogenic stimuli such as vascular endothelial growth factor (VEGF) [57–59], certain hormones (e.g. oestrogen, progesterone and insulin) [60], and shear-stress [61], NO synthesis is induced via eNOS in ECs and activates specific signalling pathways leading to tumour angiogenesis and further metastasis (Figure 2). However, NO synthesized via the action of iNOS in tumour stroma in B16 melanoma also induced the VEGF expression and angiogenesis, which implicate different roles for NO based on the tumour microenvironment, which is not the same for all types of tumours [62]. Another key player in the regulation of EMT, ECM remodelling and immune evasion in tumours is TGF-β [63]. Examining specifically the interplay between of TGF-β and NO in the regulation of angiogenesis, there is limited data available regarding cancer. Hints of potential interplay can be found from studies such as Goto et al., who showed the NO was key for the maintenance of healthy vasculature. Long-term blockade of NO resulted in upregulation of TGF-β and bFGF which led to arteriolar medial thickening and perivascular fibrosis [64]. In a diabetic wound healing model, upregulation of iNOS and ATF3 led to a significant elevation in caspase-3, -8, and -9 activity and a marked reduction in the expression of TGF-β and VEGF. This combination repressed neovasularisation and angiogenesis [65].

Furthermore, NO may have different effects on the ECs, which can be either proangiogenic or anti-angiogenic depending on its concentration and duration of exposure via cGMP signalling pathway [66]. The treatment of HUVECs with DETA-NONOate at the concentrations up-to 0.1 µM caused a 50% decrease in TSP-1 protein expression along with an increase in cell viability [67], while higher NO concentrations up-to 1 µM caused a gradual increase in the protein expression up-to 75% and cell viability. Furthermore, at 1 µM, TSP-1 gene expression in HUVECs was downregulated, but this was suppressed following the treatment with MAPK/ERK[1/2] inhibitor U0126, with the inhibition of cGMP-mediated ERK phosphorylation. Low fluxes of NO at 10 µM was responsible for proangiogenic response and cell proliferation mediated by ERK phosphorylation, which was suppressed following the treatment with exogenous TSP-1. Similarly, the treatment of HUVECs and BAECs with the slowly releasing NO-donor DETA-NONOate up-to 100 µM caused increased cell proliferation that was abrogated following the concomitant treatment with the sGC inhibitor, 1H-[1,2,4]oxadiazole[4,3-a]quinoxalin-1-one [68].

Moreover, NO-stimulated HUVEC chemotaxis at the low DETA-NONOate concentrations was inhibited after treatment with TSP-1, mediated via CD36 (Figure 2). Similar effects were due to the 3 type 1 repeats of TSP-1, but not by the trimeric N-terminal fragment (NoC1), which contains the N-domain of TSP-1. Also, The NO-induced cGMP and VEGF- levels were inhibited at 100 pM TSP-1. Also, the treatment with DEA-NONOate for fast release of NO at the concentrations up-to 100 µM stimulated the adhesion of cells, while higher concentrations caused its inhibition. The contemporary culture of cells with 10 µM DEA-NONOate and TSP-1 at a concentration higher than 22 nM abrogated the NO-stimulated cell adhesion to collagen 3D matrix. These results indicate that the cGMP signalling mediates the stimulatory effects of NO, particularly the proliferation, adhesion, and chemotaxis (or chemotaxis and chemokinesis) at low NO concentrations. However, at high concentrations, these processes are inhibited independent of cGMP and mainly through p53 and MAPK phosphatase 1 expression. Furthermore, the effects of TSP-1 were found to be through the inhibition of cGMP itself, or through inducing the cellular metabolism of NO. These observations were supported by an ex vivo study using biopsies of a pectoralis major muscle from wild type (WT) and TSP-1-null mice embedded in 3D type I collagen matrix. While DETA-NONOate at 1 and 10 µM stimulated explant outgrowth in TSP-1 null mice rather than in the WT, the higher concentrations caused inhibition of the outgrowth with a drop in tube formation and cell invasion.

Moreover, the treatment of HUVECs and human aortic vascular smooth muscle cells (VSMCs) with NO from 10 µM DEA-NONOate induced myristate-stimulated cells adhesion to collagen [69]. The VSMCs highly express CD36 with fatty acid translocase activity compared to the HUVECs. Through myristate uptake and the downstream membrane translocation, the proto-oncogene tyrosine-protein kinase (Src) is activated [70], which induces the NO synthesis (Figure 2). The original mimetic of the TSP-1 analogue, ABT-510, GDGV(DI)TRIR inhibited the cell adhesion more than ABT-510 for the same concentrations. Although this relates mainly to the binding to CD-36, there may be other receptors for both of them on these cells. Similar observations were obtained from an *ex vivo* study, where the treatment of both explanted WT and TSP1^−/−^ muscle biopsies in collagen matrices with DETA-NONOate (10 µM) caused enhanced relative vascular outgrowth in the later biopsies owing to the inhibited expression of TSP-1. However, the binding peptides to CD36, CD47 or heparin sulfate proteoglycans derived from TSP-1 inhibited this outgrowth, but NoC1 enhanced the vascular outgrowth [71]. Furthermore, the observed inhibition of angiogenesis in CD36^−/−^ muscle biopsies treated with DETA-NONOate and TSP-1, and the non-sensitivity of angiogenesis in CD47^−/−^ muscle biopsies exposed to NO to the TSP-1 treatment refers to the necessity of CD47 as a receptor for the inhibitory functions of TSP-1. These results were supported by the studies performed on CD47-null aortic smooth muscle cells (ASMCs) and HUVECs, where the ligation of CD47 was found to be essential for inhibiting the NO-induced cell proliferation and NO-stimulation of cGMP (Figure 2). However, although NO from DEA-NONOate was found to stimulate the adhesion of HUVECs and human ASMCs to collagen matrices, this was inhibited following the treatment with a group of CD36 ligands, along with inhibition of the NO-induced accumulation of cGMP in ASMCs. This indicates the importance of CD36-ligation in the inhibition of NO-signalling, but which is not essential for inhibiting the angiogenesis exerted by TSP-1 itself. Also, the enhanced proliferation of human VSMCs treated with DETA-NONOate (10 µM) was reversed in the presence of TSP-1; however, this inhibition was concentration-dependent with no differences at concentrations higher than 0.22 nM in the presence or absence of NO [72]. Furthermore, TSP-1 inhibited both the NO-stimulated cell proliferation on collagen and gelatine matrices and NO-stimulated migration on fibronectin, collagen and vitronectin. Similarly, TSP-1 inhibited the NO-induced cGMP accumulation at all tested concentrations and the cell adhesion following exposure to NO and treatment with cGMP phosphodiesterase inhibitors referring to the roles of TSP-1 is only regulating the synthesis of cGMP. Additionally, the exposure of human aortic VSMCs from WT mice to NO accompanied reduced proliferation, adhesion to collagen matrix, and cGMP accumulation compared to the NO-treated TSP null VSMCs [72].

## Influence of shear stress and mechanical properties of the ECM on NO generation and tumour angiogenesis

NO has been considered one of the important shear stress-responsive signalling molecules in ECs [57,75,76]. For instance, owing to the exposure of the ECs lining the blood vessels to laminar shear stress, such as that elicited by blood flow, increased expression of eNOS as well as the subsequent NO production has been reported [77–79]. This was found to be mediated through definite cell-matrix interactions under the stress conditions, with the alignment of the ECs in the direction of blood flow [80–82], and these findings may then translate to the cancer cells. Similar results were observed in case of cultured HUVECs under both the steady and pulsatile laminar flow at the same shear stress values; however, no effect of turbulent flow on eNOS expression and NO synthesis was observed [78]. It was found out that, under the flow effect, the actin microfilaments in the ECs are rearranged into stress fibers with parallel alignment to the flow-direction [83]. Next, the cytoskeleton may work as a second transducer for the biomechanical stimulus to the nucleus of the EC, resulting in transcriptional activation of eNOS mRNA [84]. In addition, the exposure of ovine fetoplacental artery endothelial cells to 15 dyn/cm^2^ shear stress resulted in phosphorylation of the Ser1177 residue on eNOS demonstrating one mechanism by which shear stress rapidly controls the release of NO [85]. Moreover, using D2Q9 lattice Boltzmann model for simulating the lymphatic fluid flow, the endothelium NO-release was found to be also be shear-stress dependent, which further causes dilation of the vessel and increase in the flow [86].

Relying on the properties of the ECM within the tumour microenvironment, particularly the alignment of the protein/polymer fibres [87,88], dimensions [89], pore size, and the stiffness of the matrix [90,91], the cell behaviour including its mechanical motion, transmigration and differentiation are modulated. Moreover, these features of the matrix are tuned by the cells themselves through certain signals inducing matrix degradation as well as its synthesis via the secretion of particular crosslinking factors along with specific forces generated by the cells [92]. An overview on the solid and fluid mechanics of tumours and how they affect the formation of blood vessels and cancer cell growth and metastasis has been presented by [93]. In vitro it has been shown that macrophages grown on high stiffness substrates secrete higher levels of pro-inflammatory mediators including TNF-α, NO and IL-1β than macrophages on softer substrates [94]. In endothelial cells the opposite may be true. Fels et al. showed that the nanomechanic properties of endothelial cells determines the levels of NO release, with ‘soft' cells releasing higher levels of NO than ‘hard' cells [95].

The incubation of human umbilical artery ECs with κ-elastin caused a significant increase in eNOS mRNA expression (31%) only at the elastin concentration of 2.5 µg/ml. In comparison, the concentration of 0.5% did not affect it [73]. However, neither any of these concentrations caused any changes in eNOS at the protein level, except after incubation with elastin and glucose or LDL, but not at significant levels. The treatment of co-cultured human ASMCs and human aortic ECs with different concentrations of NO released from *S*-nitroso-glutathione caused enhanced cell proliferation along with increased synthesis and deposition of elastin, HA, and glycosaminoglycans by the SMCs within the cell matrix layers [96]. The treatment of BAECs with tropoelastin caused a significant increase in NO production proportional to the protein concentration, with a maximum response at five µM; however, this enhancement was reduced after L-NAME treatment [74]. Furthermore, the non-significant changes in NO production following the treatment with the iNOS inhibitor 1400W, and the significant inhibition of NO synthesis following silencing of eNOS with specific siRNA refer to the role of tropoelastin in inducing NO synthesis through eNOS only. This was confirmed by western blotting: the levels of phosphorylated Akt and eNOS were enhanced in the case of tropoelastin and reduced with the Akt inhibitor, wortmannin proving that tropoelastin works via the PI3K-specific pathway in enhancing the NO production in ECs.

**Figure 3.**
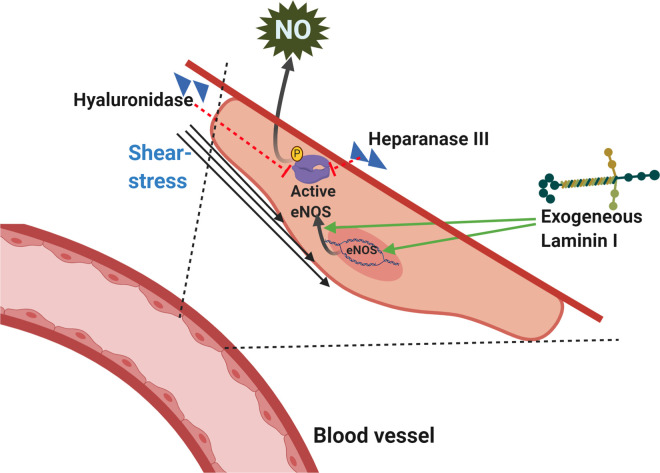
The stimulated expression of eNOS and NO synthesis in endothelial cells under shear stress and the effects of different components of the ECM. The mRNA and protein expression of eNOS is stimulated under the influence of shear stress leading to enhanced NO production, which is promoted in the presence of exogenous laminin [98,99]. The treatment of the arteries with active hyaluronidase as well as heparanse III cause inhibition of the flow-induced NO-production [100]. The scheme was created in Biorender.com.

BAECs monolayers exposed to physiological shear stress exhibited alignment in the direction of flow with elongation following the treatment with intact glycocalyx [97]. However, during the treatment with either heparanase III for degradation of heparin sulfate (HS) or with silencing of glypican 1, the HS core protein caused blocked shear-induced eNOS activation with decreased expression of only the phosphorylated eNOS (Figure 3). Furthermore, the knockdown of syndican-1 by specific shRNA did not have significant effects on the flow-induced phosphorylated eNOS expression. These effects were only significant under the shear-stress conditions, with no changes in expression of proteins from the static cultures. These results, in addition to the preserved ECs remodelling in response to flow following glypican one knockdown only indicate its functionality in mediating the shear-induced eNOS activation. The expression levels of eNOS gene in ECs isolated from the porcine aorta and cultured in coated plates with either laminin I, Col 1, or fibronectin under a shear stress of 16 dyne/cm^2^ for 6 h showed almost no change, moderate, and twofold increase, respectively [98]. This was alongside an increase in eNOS protein expression and nitrite production in the case of laminin I coating. However, the addition of the peptide YIGSR, deduced from the B1 chain of laminin caused inhibition of that expression, and it was observed previously to cause attenuation of tumour invasion *in vivo* [99].

Furthermore, laminin I added to cells under static conditions did not change eNOS expression, referring to its modulating roles for the response of a vessel to the applied mechanical force mediated by shear stress sensitivity of cells in terms of eNOS expression involved in tumour invasion. Moreover, the treatment of isolated canine femoral arteries with hyaluronidase resulted in a significant decrease in the flow-induced NO production, but this flow was not affected in the case where heat-inactivated hyaluronidase was used [100] (Figure 3). This bears out to the importance of Hyaluronan as one of the main components of the glycocalyx layer in the regulation of the shear force of flowing blood in arteries, which induces NO synthesis in the ECs.

For metastasis of cancer cells to occur, degradation of ECM is an essential step, which was found to be facilitated by invadopodia formation, with the functions of these membrane protrusions found to be dependent on its lipid rafts and caveolae components [101,102]. These caveolae are subcellular structures with ventral membrane protrusions and within the tumour microenvironment, they were found to be sensed by the two mechanical stimuli: ECM stiffness and shear stress [103]. The response starts with a rapid flattening of the caveolar invagination, followed by disassembly and release of caveolin-1 as one of its main protein components [104]. For instance, in breast cancer, caveolin-1 was found to cololocalize with MT1-MMP at the invadopodia mediating breast cancer cell invasion [105]. Moreover, caveolin-1 was found to be responsible for the formation of invadopedia and breast cancer metastasis *in vivo* mainly via the induction of PI3K-specific pathway [106].

Interestingly, a negative regulation of NO synthesis was observed in cells expressing caveolin-1, where a particular caveolin-1 scaffolding domain was found to interact with eNOS causing inhibition of its enzymatic activity [107–109]. Furthermore, vascular perfusion was found to enhance the expression and activity of eNOS within the caveolae of the luminal cell surface of the endothelium indicating a role for caveolae in the transduction of the mechanical stimuli and regulation of eNOS activity [110].

## Influence of the ECM and NO on tumour angiogenesis in different tumour types

### Melanoma

The exposure of B16 melanoma explants to NO from 10 µM DETA-NONOate stimulated the vascular cell outgrowth from the explants in the 3D collagen matrix. However, the TSP-1 analogue ABT-510 inhibited this stimulation [69]. Furthermore, DEA-NONOate resulted in a rapid decrease in tumour blood flow within the abnormal vascular network of the tumour and increased flow in the distant vascular networks, in murine B16F10 and human MDA-MB-435 melanoma cell lines xenografts [111]. However, in tumours with overexpressed TSP-1, these blood flow-changes were attenuated with a decrease in the total vessel area and the fluorescence signal of CD31- positive vessels. This refers to the loss of perfused vessels and suggests that the TSP-1 based drugs function mainly as inhibitors of the angiogenesis, which resist the NO-induced angiogenic cascades in melanoma, with limited effects on blood flow [111].

### Colorectal cancer

NO also plays an essential role in the promotion of colorectal cancer growth and angiogenesis. iNOS mRNA, protein and activity are elevated in colorectal cancer compared to the healthy tissues and were higher in metastatic than in non-metastatic tumours with a corresponding increased cGMP production [112,113]. The composition of the ECM in colorectal cancer along with the effects of the different proteins on cancer cell metastasis have been discussed previously [114,115]. Furthermore, the composition of the ECM depends on the stage of cancer, with less organized matrix correlated with higher proliferation of the cancer cells [116,117].

The treatment of LS174T colonic adenocarcinoma cells with SNP (10 mM) caused enhanced mucin secretion and increased levels of nitrite, indicating released NO [118]. A significant decrease in cell viability accompanied this. The NO scavenger myoglobin reversed these effects. In contrast, both the induction of intracellular NO synthesis by treatment with L-arginine or the inhibition with L-NG-monomethyl Arginine citrate did not significantly affect the secretion of mucin. This indicates that intrinsic sources of NO synthesized via the eNOS and nNOS probably do not play a role in modulating mucin release, and in protection against damage. Instead, exogenous sources of NO enhance the release of non-mucin components before epithelial cell damage, with the corresponding mucosal protective functions. For instance, the IHC staining of Muc1 was higher in patients with metastatic tumours than in those without metastasis, with the lowest levels in normal colon mucosa samples [119]. In contrast, following the induction of initial colon carcinogenesis by dimethylhydrazine in mice, the number of goblet cells responsible for the secretion of Muc2 was found to decrease referring to their roles in the inhibition of colon carcinogenesis [120]. This was correlated with increased NO levels in both the proximal and distal colon as a measure of the oxidative stress following carcinogenesis induction. In another histological study, iNOS expression was increased in colorectal cancer tumours compared to healthy controls and was also elevated in biopsies from patients with inflammatory bowel disease (IBD). TSP-1 expression (mainly cytoplasmic), was intermediate between the healthy and IBD groups but not statistically significant. TSP-1 expression was positively correlated with iNOS expression [121]. These observations refer to a positive correlation between the expression of NOS and TSP-1 within the colon cancer tissue, while this correlation does not exist with mucin expression. However, only the exogenous NO caused increased secretion of mucin within the tissue with co-operative roles played in modulating the tumour growth.

### Breast cancer

As with its effects on the other cancers and based on the tumour microenvironment and its concentration, NO may act as either an inducer for tumour cell metastasis and tumour progression or may ameliorate it [12,122]. The ECM components induced during breast cancer have been previously discussed [123]. NO production within the breast tissue acts as a link between the architecture and function of breast tissue, and its involvement in breast cancer has been studied, and in particular, its roles in modulating cell-ECM interactions. The variable number tandem repeats four a/b polymorphism in eNOS has been found to be associated with the development of breast cancer, indicating a role for NO [124]. The treatment of the MCF-7 breast cancer cell line with curcumin exhibited decreased cell proliferation, and these cytotoxic effects were enhanced in the presence of either fibronectin or collagen accompanied by decreased NO generation [125].

Exposure of non-malignant MCF10A breast cells to laminin (LN5) resulted in increased P-53 (pSer20-p53) and phosphorylated NOS-1 (Ser1417 phosphorylation) [126], which was reversed by the NOS inhibitor L-NAME. Furthermore, following treatment of MCF-10A, non-malignant S1 and malignant T4-2 cells with LN5, the nitrite levels increased in MCAF-10A and S1 cells, but not in T4-2 cells. In contrast, Collagen 1 (Col 1) could induce NO synthesis in S1 and MCF10A cells. However, the treatment of cells with LN1-rich ECM gels (lrECM) caused increased levels of the fluorescently detected intracellular NO, which peaked at 1 h in the case of S1 and MCF10A cells but remained low in T4-2 cells. Intriguingly, T4-2 cells reverted to a non-malignant phenotype in the presence of laminin, and S1 cells showed intense staining for S-nitrosocysteine (SNOC) as an indicator for NO production, whereas T4-2 had weak staining relating to to the recovery of the cell's ability to produce NO following the phenotypic reversion. Moreover, both MCF10A and S2 cells had disorganized proliferative structures in IrECM following the inhibition of NO production by L-NAME treatment referring to the induction of malignant behaviour, while the phenotypic reversion was induced after treatment with SNAP. These findings refer to the importance of NO production mediating p53 activation in response to IrECM towards the formation of mammary acini and downregulation of MMP-9 expression for the further inhibition of laminin degradation.

Furthermore, in a study by Ricca et al. 2018 [127], a short-time compression was applied to both T4-2 and S1 cells embedded in IrECM gel. After culturing for ten days, mechanical reversion happened to the cells with the formation of acinar-like structures with the restoration of the coherent rotation in the malignant cells. This was correlated with a reduction in the size of colonies from T4-2 cells under compression to be similar to that of the S1 acini. Moreover, the detected NO became twofold following the compression of these cells for 30–60 min, referring to the induction of NO synthesis. Following the inhibition of NO synthesis by L-NAME, the mechanical reverting was absent with no significant reduction in colony size. However, the mechanical reverting and the development of the same phenotypically healthy program observed by [92] was promoted in the case of SNAP. Furthermore, the culture of the breast cancer progression series premalignant AT1, malignant CA1d and DCIS cells, able to form ductal carcinoma *in situ*, in IrECM gel caused reduced production of NO and nitrite along with reduced levels of the cytosolic SNOC compared to MCF10A cells [128]. However, the levels of nNOS and eNOS remained high in all cell lines, with a reduction in the levels of iNOS; both nNOS and eNOS share equally in maintaining the basal NO levels in MCF10A cells. Furthermore, in the malignant cell lines, the levels of superoxide and ROS were higher than that in controls and MCF10A with a dramatic reduction in the levels of tetrahydrobiopterin (BH4) responsible for the dimerization of each of the two NOS monomers, thus inhibiting the synthesis of NO, with a reduction in its basal levels during cancer progression.

The IHC analysis of 185 invasive breast cancer lesions done by Karihtala et al. 2007 [129] had the following results: (i) the HA levels in the stroma of all lesions were moderate-to-high and were correlated with sizeable primary tumour size and higher histological grade, with associated high nNOS expression in the cancer cells. (ii) The cancer cell-associated HA levels were negative-to weakly positive, with an inverse correlation to the levels of nitrotyrosine, but without significant association between these levels of staining and the large tumour size. Moreover, the increased synthesis of NO activated the HA synthesis and its stromal accumulation. However, the decreased levels of the cell-associated HA relate to the partial degradation by the ONOO^-^ ions from the intracellularly generated NO and O_2_^−.^ molecular fragments, correlated with the nitrosation of tyrosine.

## Conclusions

In spite of the recent progress in studying the effects of the mechanical properties of the ECM on NO production in different diseases, limited progress has been achieved in addressing its specific role in cancer metastasis. For instance, the role of NO in bone mechano-transduction has been demonstrated *in vivo*, where the treatment of rats with L-NAME caused a reduction in mechanically-induced bone formation in the tibiae [130]. Furthermore, a decrease in NO levels was observed following the culturing of primary bone cells in medium from adaptive tumour cells in response to low-magnitude, high-frequency vibrations along with a decrease in the degree of mineralization with variation in cell cytoskeletal arrangement and matrix stiffness [131]. More studies are recommended to address the mechanisms by which the tumour cell components are sensed by the ECM stiffness, and how this is translated to the modulation of NO synthesis. Furthermore, the mechanisms by which shear stress induces NOS activation, mediated by lipid rafts and caveloae, may be similar to the effects of ECM stiffness and further investigations of that are essential. Further studies are needed in different cancer types to increase our understanding of how NO contributes to the formation of the tumour microenvironment. Understanding this will help us to better design new anti-cancer therapeutic strategies targeting the tumour microenvironment and tumour angiogenesis.

## Perspectives

- Importance of the field: Tumour angiogenesis and growth are multifactorial processes, with a significant role played by the ECM proteins and enzymes in their regulation. NO, through its dichotomous effects, regulates these processes either through activating or inhibiting specific ECM proteins, with mutual effects of these proteins on NO production within different tumours.
- Summary of the current thinking: NO's modulation of tumour growth is dependent on NO concentration & NOS activity level, duration of exposure, physical localization of NOS enzymes. This, together with the composition of the tumour ECM, mediates the effects of NO on tumour progression.
- Future directions: Studying the interactions between NO and ECM components will improve our understanding of relevant changes within the tumour microenvironment, which drive further angiogenesis and tumour growth. Moving forward, these findings will guide the development of new and more effective therapeutics for cancers, and increase our understanding of the biology of aggressive tumours.

## Abbreviations

ASMCs: aortic smooth muscle cells
BAECs: bovine aortic endothelial cells
cGK-1: cGMP-dependent protein kinase
cGMP: buanosine 3′,5′-cyclic monophosphate
DEA-NONOate: biethylamine NONOate
DETA-NONOate: diethylenetriamine NONOate
ECs: endothelial cells
eNOS: endothelial nitric oxide synthase
ERK: extracellular signal-regulated kinase
GSNO: *S*-nitroso-glutathione
GTP: guanosine triphosphate
HNSCC: head and neck squamous cell carcinoma
HUVECs: human umbilical vein endothelial cells
iNOS: inducible nitric oxide synthase
L-NAME: N(gamma)-nitro-L-arginine methyl ester
MAPK: mitogen-activated protein kinase
MMP: matrix metalloproteinase
MT1-MMP: membrane Type 1-MMP
nNOS: neuronal nitric oxide synthase
NoC1: trimeric N-terminal fragment
PKG: protein kinase G
sGC: soluble guanylyl cyclase
SNAP: S-nitrosoN-acetylpenicillamine
SNOC: S-nitrosocysteine
SNP: sodium nitroprusside
TIMP: tissue inhibitor of metalloproteinase
TSP-1: thrombospondin
VEGF: vascular endothelial growth factor
VSMCs: vascular smooth muscle cells
WT: wild type
